# Knockdown of SESN2 Exacerbates Cerebral Ischemia–Reperfusion Injury Through Enhancing Glycolysis via the mTOR/HIF‐1α Pathway

**DOI:** 10.1111/cns.70314

**Published:** 2025-03-03

**Authors:** Zhihui Wang, Yingao Huang, Yonggang Zhang, Hua Zhu, Mohammad Rohul Amin, Ran Chen, Lijuan Gu, Xiaoxing Xiong

**Affiliations:** ^1^ Department of Neurosurgery Renmin Hospital of Wuhan University Wuhan China; ^2^ Central Laboratory Renmin Hospital of Wuhan University Wuhan China

**Keywords:** ischemia–reperfusion injury, metabolic reprogramming, p‐mTOR/HIF‐1α, SESN2

## Abstract

**Aim:**

Reprogramming of glycometabolism plays a crucial role in the pathogenesis of cerebral ischemia–reperfusion injury (CIRI). Sestrin2 (SESN2), a sensor upstream of the mTORC1, is closely related to glycometabolism. However, the effect and mechanism of SESN2 in CIRI are unclear. The goal of this research was to explore the effect of SESN2 on CIRI and its potential mechanisms related to glycometabolism.

**Methods:**

Lentiviral vectors carrying SESN2 shRNA (Lenti‐SESN2) or negative NC virus (Lenti‐GFP) or rapamycin (mTOR inhibitor) were employed in the oxygen–glucose deprivation/reoxygenation (OGD/R) model and in the middle cerebral artery occlusion (MCAO) mice. In all, 3 days after I/R, neurological deficit scores and infarct size were assessed. The glycolysis and SESN2 levels were determined by RT‐qPCR, Western blots, and immunofluorescence staining. Lactate levels were detected by a lactate assay kit, and the expression of the p‐mTOR/HIF‐1α signaling pathway was measured by immunofluorescence staining and protein blotting.

**Results:**

Local SESN2 deficiency in brain tissue increased the infarct size and reduced neurological scores 3 days after I/R. Moreover, the results showed that local SESN2 deficiency in brain tissue increased the expression of glycolysis‐related proteins, including HK2, PFKM, PKM1, PKM2, and GLUT1. The lactate assay kit showed that local SESN2 deficiency in brain tissue increased lactate levels. In addition, local SESN2 deficiency in brain tissue improved the expression of the p‐mTOR/HIF‐1α pathway. However, rapamycin (RAP) treatment reversed these results, suggesting that SESN2 may influence IS injury by regulating glycometabolism via p‐mTOR/HIF‐1α pathway regulation. SESN2 knockdown in BV2 cells improved the glycolysis levels and the expression of the mTOR/HIF‐1α pathway in the OGD/R model in vitro, but RAP treatment can also reverse these results.

**Conclusions:**

Knockdown of SESN2 in MCAO mice increased the expression of the p‐mTOR/HIF‐1α pathway, which increased glycolysis and lactate levels and, in turn, affected IS injury.

## Graphical abstract

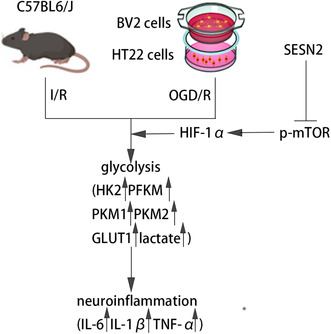

## 1 Introduction

One of the major causes of disability and death is ischemic stroke (IS) [1, 2]. Current clinical treatment measures place emphasis on restoring the perfusion of blood flow in the brain to salvage the ischemic penumbra [3]. The ischemic penumbra is an area of reversible damage under hypoperfusion because the blood flow of the penumbra is reduced by 20%–40%, which surrounds the core of irreversible damage, but over time, if there is no treatment within a limited time window, the ischemic penumbra will evolve into the irreversibly damaged core [1, 4]. Nevertheless, once blood flow recovers, significant injury occurs, known as I/R injury, which triggers a rapid series of neuropathological events, consisting of dysregulation of ion homeostasis, abundant ROS generation, and oxidative and nitrosative stress, ultimately leading to neuronal cell death [5]. It is known that in the mammalian brain, glucose is the main source of energy and glucose metabolism is closely related to brain pathophysiology and function [6]. Under ischemic and hypoxic conditions, the brain undergoes a reprogramming of glycometabolism (transition from oxidative phosphorylation under normoxia to glycolysis under hypoxia) to satisfy high energy requirements [5, 6]. However, previous studies showed that glycolysis in the ischemic penumbra increased significantly under normoxia after restoration of cerebral blood perfusion [7, 8]. This implies that brain tissue in the penumbra undergoes different reprogramming of glucose metabolism (transition from oxidative phosphorylation under normoxia to glycolysis under normoxia) after I/R. However, with the increase of glycolysis levels, the production of lactate will also increase, which is closely related to ischemic diseases. Inhibition of stroke‐induced hyper‐activated glycolysis and lactate production has some neuroprotective effects [7, 8]. Therefore, the conversion of brain glucose metabolism from glycolysis to oxidative phosphorylation is a critical strategy for the treatment of IS and is also critical for protecting the neurons in the peri‐ischemic region. In addition, glucose metabolism reprogramming is an important pathological mechanism in IS. Although disturbed glucose metabolism is a common neuropathologic feature of cerebral I/R injury, the potential mechanisms remain to be clarified.

Sestrin2 (SESN2), a member of the Sestrin family, is expressed in a wide range of organisms, including humans, and can be induced by multiple stress events (hypoxia, energy deficiency, and oxidative stress) to exert powerful antioxidant effects. Moreover, SESN2 can maintain metabolic homeostasis by regulating the rapamycin pathway (mechanistic target of rapamycin [mTOR]) [9]. Some reports have found that the mTOR/HIF‐1α pathway is closely related to the reprogramming of glucose metabolism in a variety of diseases [10, 11, 12]. In addition, SESN2 is strongly associated with a variety of metabolism‐related diseases, including diabetes and cancer [9]. Many researchers have confirmed that SESN2 plays a protective role in a variety of ischemic diseases [13, 14, 15, 16, 17, 18]. Moreover, it has been confirmed that SESN2 can interact with OXPHOS complexes to protect mitochondrial function, which in turn maintains metabolic homeostasis to reduce cardiac I/R damage [14]. SESN2 can inhibit ROS production by activating AMPK to alleviate cardiac I/R injury [15]. Intranasal administration of recombinant human SESN2 (rh‐SESN2) provides neuroprotective effects in hypoxic–ischemic encephalopathy by conditioning the AMPK/mTOR pathway in rat newborns [17]. In addition, SESN2 regulates microglial cell polarization through the mTOR signaling pathway to reduce neuron apoptosis and neuroinflammation after I/R [19]. Nevertheless, it is uncertain whether SESN2 is associated with glucose metabolism reprogramming in IS, and the specific mechanisms are also unclear.

In this research, we investigated whether SESN2 knockdown leads to neurological damage in an OGD/R model and in MCAO mice. Then, we investigated whether the neurological damaging effects of the knockdown of SESN2 after IS were related to the interference of glycolysis and a potential signaling pathway.

## 2 Methods

### 2.1 Animals

The mice (C57BL/6J, 25–30 g, male, 8–10 weeks) were purchased from Wuhan University Center for Animal Experiments. They were maintained under certain disease‐free conditions at the Animal Experiment Center of Renmin Hospital of Wuhan University. At least 7 days before the experiments, the mice were housed at controlled humidity (60%–70%) and temperature (24°C–26°C) with half daylight and half nighttime cycling.

### 2.2 Drug Administration

Rapamycin (553,211; Sigma‐Aldrich, MO, USA) was stored at −20°C under desiccated (hygroscopic) protect from light. Rapamycin was administered (10 mg/kg, i.p.) immediately after stroke, as previously reported [20, 21].

### 2.3 Lentivirus Administration

As previously described, GeneChem (Shanghai, China) constructed lentiviral vectors carrying Lenti‐SESN2 [22], targeting mouse SESN2. Lenti‐SESN2 was injected into the left cerebral hemisphere according to previously described methods [23, 24]. Briefly, we performed three injections: Points 1 and 2, 3.0 mm lateral (left), 0.8 or 1.9 mm posterior to the bregma, 2.0 mm deep, and Point 3, 3.0 mm lateral (left), 0.3 mm anterior to the bregma, 2.0 mm deep. Concentrated lentivirus (1.4 μL per point, 1×10^9^ transducing units/mL) was injected at a constant rate (10 min/point). Mice were subjected to sham or MCAO procedures 5 days after injection.

### 2.4 MCAO Model

After 1 week of acclimatization feeding, the mice were used to build the MCAO model as described previously [25, 26]. Briefly, under isoflurane (2.5% isoflurane in O_2_) anesthesia, after the mouse's neck skin was dissected, ligation of the left middle cerebral artery was performed using a 6.0‐mm nylon monofilament (Doccol, Corp., Redlands, CA, USA). One hour after ischemia, the nylon monofilament was removed to establish I/R. Sham group mice underwent the same operative procedure but without monofilament ligation.

### 2.5 Infarct Volume Measurement

After 72 h of MCAO, an overdose of isoflurane was used to euthanize the mice, and then the mice were decapitated. The brains were dipped in PBS (4°C) for 0.25 h and then cut into 2‐mm coronal slices. The slices were instantly stained by incubation in 2% 2,3,5‐triphenyltetrazolium chloride (TTC) for 0.25 h at room temperature. The infarct volume (percentage of right hemisphere volume) was assessed by using ImageJ (NIH, MD, USA) according to previously reported methods [27, 28].

### 2.6 Assessment of Neurological Deficits

Three days after MCAO, neurological deficit scores were evaluated in a blinded manner according to the neurological grading scale based on previously reported values: from 0 (*without observable neurological deficit*) to 4 (*no spontaneous motor activity, impaired consciousness*) [26].

### 2.7 Immunofluorescence Staining

Three days after MCAO, an overdose of isoflurane was used to euthanize MCAO mice and sham treatment mice. The mice were then perfused with PBS (4°C), followed by perfusion with 10 mL of 4% paraformaldehyde according to previously published studies [21, 27]. The mice were decapitated, and their brains were immersed in 4% paraformaldehyde for 3 days and then cut into 50‐μm slices. The slices were dipped in 0.3% Triton X‐100 for 30 min and then were washed two times; next, after 1 h of being closed using blocking buffer (5% fetal bovine serum), the slices were incubated overnight at 4°C with the following primary antibodies: anti‐SESN2 (1:100; Proteintech Group, 10795‐1‐AP), anti‐IBA1 (1:100; ab283342, Abcam, Cambridge, UK), anti‐NeuN (dilution 1:100; ab104224, Abcam, Cambridge, UK), anti‐p‐mTOR (1:100; Proteintech Group, 67778‐1‐Ig), anti‐HIF‐1α (1:100; Proteintech Group, 20960‐1‐AP), and anti‐PKM1 (1:100; Proteintech Group, 15821‐1‐AP). The slices were washed three times, and then the Alexa 488 coupled antibody (1:500; ANT024, Millipore, Billerica, MA) or Alexa 594–coupled antibody (1:500; ANT030, Millipore, Billerica, MA) was used to stain the sections for 1 h at room temperature. After being washed three times, the nucleus was stained by using DAPI. A fluorescence microscope (Olympus Optics, Japan) was used to observe the sections. Five different regions of interest (in the peri‐ischemic region) were assessed in each group of five mice. The evaluators used ImageJ to quantify the immunoreactive cell counts in the ischemic brain in a blinded manner.

### 2.8 OGD/R In Vitro

Prior to the induction of OGD/R injury, cultured cells were washed twice and kept in glucose‐free Dulbecco's modified Eagle's medium (DMEM). Then, cells were placed in a triple gas incubator containing 94% N_2_, 5% CO_2_, and 1% O_2_ at 37°C for 6 h to induce OGD/R injury. Cells were then recovered with DMEM and under normoxic conditions for 1 day according to what was described previously [29]. The control group was rinsed twice and cultured in DMEM without hypoxia.

### 2.9 In Vitro Drug Administration and Lentivirus Administration

BV2 cells were inoculated in six‐well plates in the complete medium. After reaching 20%–30% confluence, BV2 cells were treated with Lenti‐SESN2 or Lenti‐GFP. After 72 h of transfection, puromycin (1 μg/mL) was added to screen. The cells that were not successfully infected were killed by puromycin. After all the BV2 cells were successfully infected with lentivirus, the concentration of puromycin was reduced to 0.5 μg/mL. Besides, BV2 cells subjected to different treatments were treated with rapamycin (20 nM) for 1 h before the induction of OGD.

### 2.10 Coculture Experiment and Cell Viability Measurement

HT22 cells were cultured in the lower room of a six‐well Transwell plate, and BV2 cells subjected to different treatments were cultured in the higher room of the six‐well Transwell plate. After 24 h of co‐culturing in the complete medium in an incubator (37°C, 5% CO_2_), they were used to perform the OGD/R damage. After 24 h of OGD/R, the HT22 cells that received different treatments were isolated and plated in 96‐well plates. Then CCK‐8 assays were used to assess the viability of HT22 according to the instructions. Absorbance values at 450 nm were measured with an enzyme labeler. Cell viability is shown as fold change compared to the control group or OGD/R group.

### 2.11 Measurement of Lactate

Three days after IS or 24 h after OGD/R, lactate content was assayed from BV2 cells that received different treatments and ipsilateral brain tissue: lactate assay kit (BC2235; Solarbio) was used, and absorbance was measured directly at 570 nm using an enzyme marker according to the instructions. Samples were previously analyzed, and we tested several samples to determine the applicable loading amount, ensuring that the readings were on a standard curve. The lactic acid content of the samples was calculated according to the appropriate formula: lactic acid content (μmol/L) = (absorbance of sample−absorbance of blank)/(absorbance of standard−absorbance of blank)×concentration of standard (μmol/L)×number of dilutions. The levels of lactate are shown as fold change compared to the sham group, control group, MCAO group, or OGD/R group.

### 2.12 Quantitative Real‐Time PCR

Mouse brains were obtained after using an overdose of isoflurane to euthanize mice at 3 days after MCAO, and we used the ipsilateral hemisphere. After 72 h of MCAO or after 24 h of OGD/R, total RNA was extracted from the tissues in the ischemic brain and BV2 cells by using TRIzol reagent (#9109; TaKaRa, Shiga, Japan) according to the instructions, and then was reverse transcribed to cDNA. PCR reverse primers are listed in Table 1. Relative mRNA expression levels were quantified by normalizing to β‐actin levels. Gene expression levels are shown as fold change compared to the sham group, control group, MCAO group, or OGD/R group.

**TABLE 1:** Primers for RT‐PCR.

Genes	Primers (5′–3′)
SESN2	
Forward	TCCGAGTGCCATTCCGAGAT
Reverse	TCCGGGTGTAGACCCATCAC
HK2	
Forward	TGATCGCCTGCTTATTCACGG
Reverse	AACCGCCTAGAAATCTCCAGA
PKM1	
Forward	CGTCCGCAGGTTTGATGAGA
Reverse	TTCAAACAGCAGACGGTGGA
PKM2	
Forward	GCCGCCTGGACATTGACTC
Reverse	CCATGAGAGAAATTCAGCCGAG
GLUT1	
Forward	CAGTTCGGCTATAACACTGGTG
Reverse	GCCCCCGACAGAGAAGATG
PFKM	
Forward	TGTGGTCCGAGTTGGTATCTT
Reverse	GCACTTCCAATCACTGTGCC
Actin	
Forward	GGCTGTATTCCCCTCCATCG
Reverse	CCAGTTGGTAACAATGCCATGT
IL‐1β	
Forward	CACCTCTCAAGCAGAGCACAG
Reverse	GGGTTCCATGGTGAAGTCAAC
IL‐6	
Forward	GGTCCAGTTGCCTTCTCCC
Reverse	GCAACAAGGAACACCACGG
TNF‐α	
Forward	GACGTGGAACTGGCAGAAGAG
Reverse	TTGGTGGTTTGTGAGTGTGAG

### 2.13 Western Blot (WB) Analysis

Three days after IS or 24 h after OGD/R, total proteins were extracted from BV2 cells that received different treatments, HT22 cells, and ipsilateral brain tissues by using RIPA lysis according to a previous study (34). The samples were then separated by electrophoresis on gels and transferred to a PVDF membrane. Then the 5% fetal bovine serum was used to block membranes for 60 min, and then they were incubated with anti‐SESN2 (1:1000; Proteintech Group, 10795‐1‐AP), anti‐HK2 (1:1000; Proteintech Group, 22029‐1‐AP), anti‐PFKM (1:1000; Proteintech Group, 55028‐1‐AP), anti‐PKM1 (1:1000; Proteintech Group, 15821‐1‐AP), anti‐PKM2 (1:1000; Proteintech Group, 60268‐1‐Ig), anti‐GLUT1 (1:1000; Proteintech Group, 66290‐1‐Ig), anti‐mTOR (1:5000; Proteintech Group, 66888‐1‐Ig), anti‐P‐mTOR (1:1000; Proteintech Group, 67778‐1‐Ig), anti‐CL‐caspase‐1 (1:1000; 89332, CST, MA, USA), or anti‐HIF‐1α (1:500; Proteintech Group, 20960‐1‐AP) antibody at 4°C overnight. The membranes were also incubated with an HRP‐labeled secondary antibody for 60 min at room temperature. Images were assessed with the Bio‐Rad Imaging System. Afterward, the optical intensity of each protein band was analyzed and normalized to the optical density of the β‐actin band using ImageJ. The expression levels of proteins are shown as fold change compared to the sham group, control group, MCAO group, or OGD/R group.

### 2.14 Statistical Analysis

We used GraphPad Prism software (Version 8.0) to analyze data. We used one‐way analysis of variance (ANOVA) to analyze the data from different groups and Student's t tests to analyze the data from two groups. The results were expressed as the mean ± SD. *p* < 0.05 denotes significance.

## 3 Results

### 3.1 SESN2 Expression and Glycolysis Are Elevated 3 Days After I/R

**FIGURE 1:**
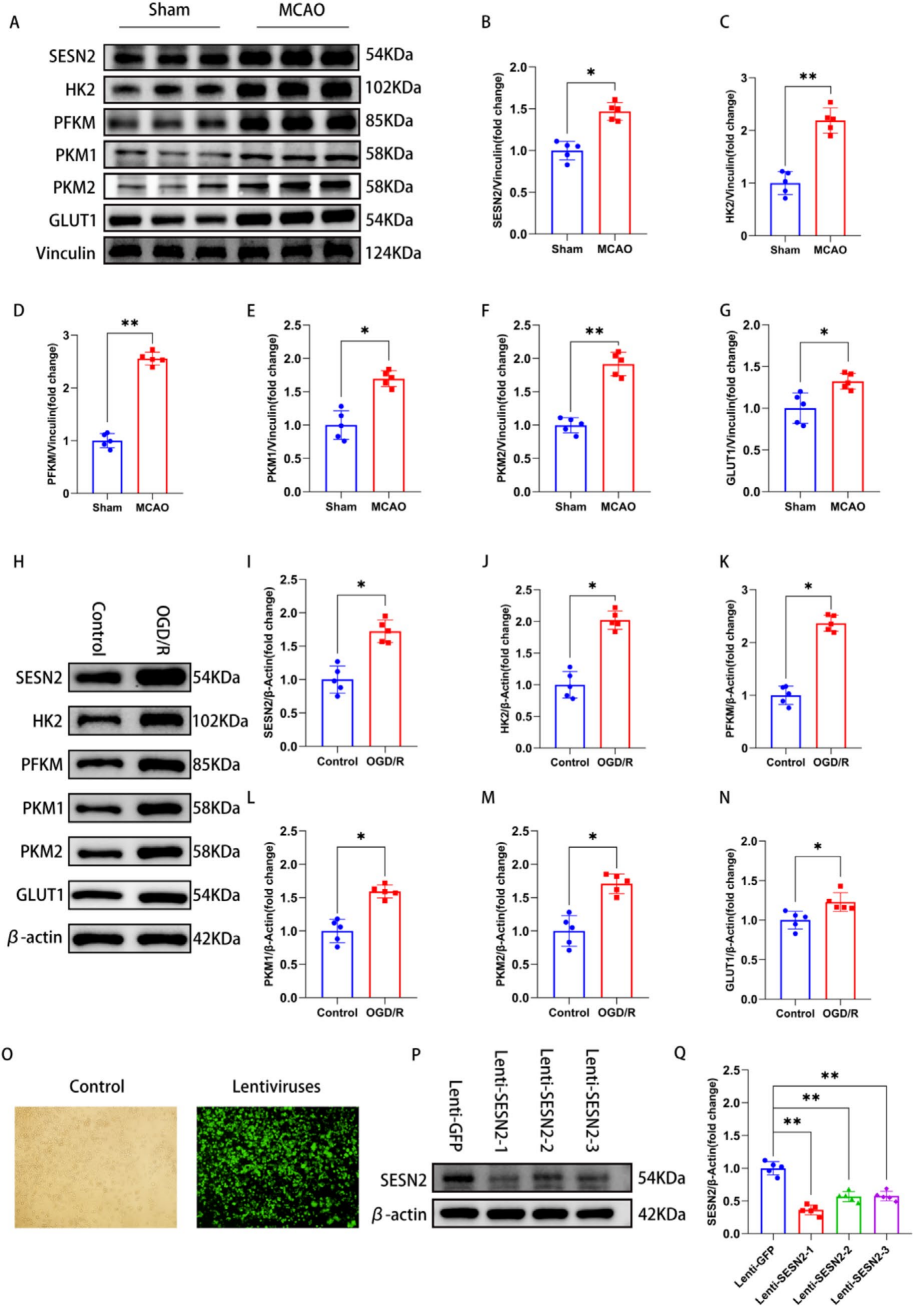
The expression of SESN2 and glycolysis after 72 h of stroke. (A) Western blot images and (B–G) quantitative analysis illustrated that the levels of SESN2, HK2, PFKM, PKM1, PKM2, and GLUT1 were elevated in ischemic brain after 72 h of stroke. (H) Western blot images and (I–N) quantitation illustrated that the levels of SESN2, HK2, PFKM, PKM1, PKM2, and GLUT1 were increased in the BV2 cells after 1 day of OGD/R. (O) All the BV2 cells were successfully infected with the lentivirus. (P) Representative Western blot images and (Q) quantification of SESN2. *n* = 5. *p < 0.05, **p < 0.01. Means ± SD.

Brain metabolism undergoes multiple metabolic reprogramming after stroke, one of the more important of which is glucose metabolic reprogramming [5, 6]. First, at 72 h after stroke, we detected the expression of SESN2 and glycolysis‐related proteins in the peri‐ischemic region by WB. The results illustrated that the SESN2, HK2, PFKM, PKM1, PKM2, and GLUT1 levels were elevated at 3 days after I/R (Figure 1A–G), indicating that glycolysis was increased after I/R. It is reported that microglial activation is accompanied by the transition of oxidative phosphorylation to glycolysis in IS [30]. To further validate the above results, we used BV2 cells for OGD/R modeling in vitro. WB results illustrated that the SESN2, HK2, PFKM, PKM1, PKM2, and GLUT1 levels were elevated 24 h after OGD/R (Figure 1H–N). These results indicated that OGD/R treatment can induce an increase in glycolysis in BV2 cells.

### 3.2 SESN2 Knockdown Promoted Glycolysis and Activated the mTOR/HIF‐1α Pathway in BV2 Cells 24 h After OGD/R

**FIGURE 2:**
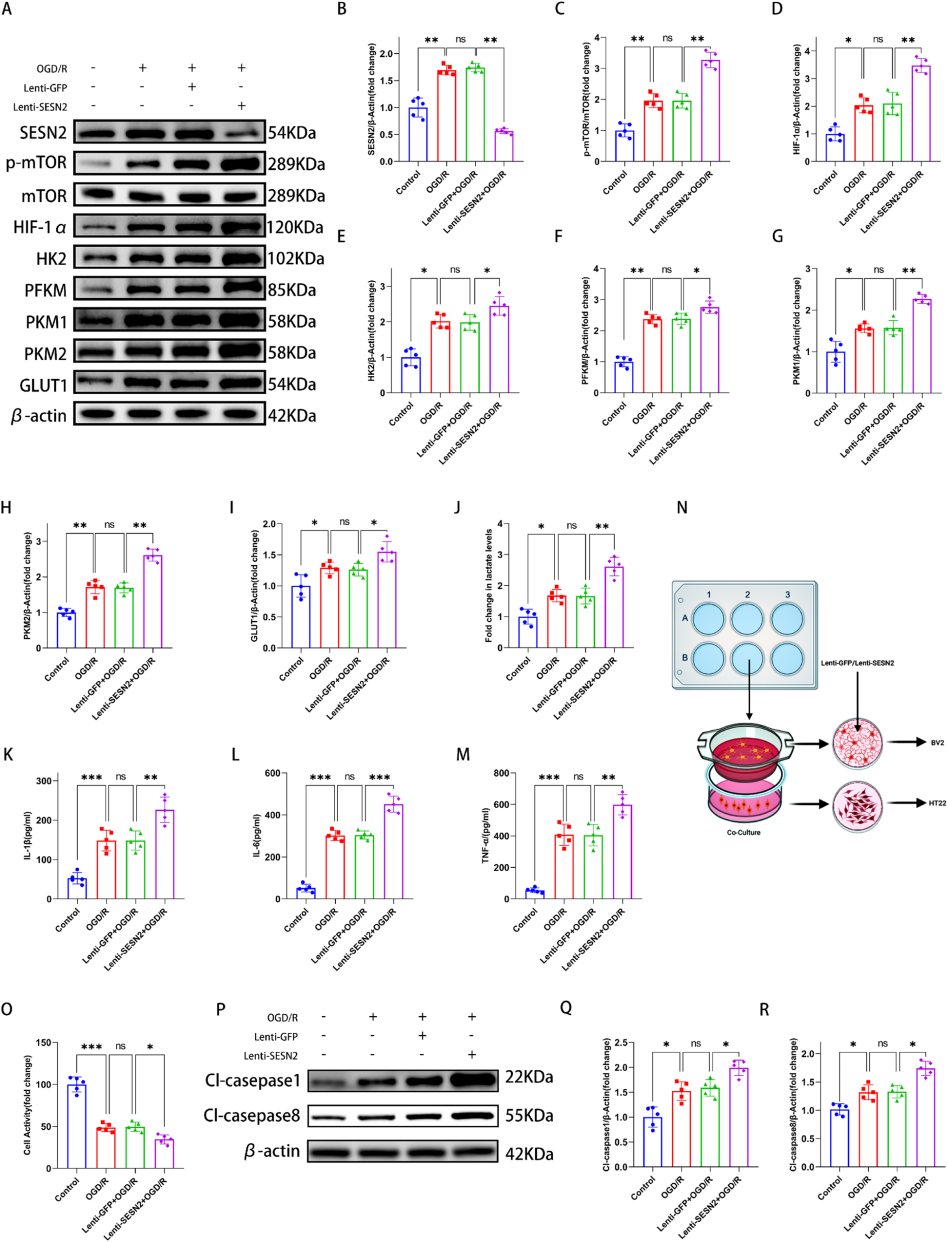
Effect of SESN2 knockdown on glycolysis‐related proteins expression and p‐mTOR/HIF‐1α pathway expression in BV2 cells after 24 h of OGD/R. (A) WB strips indicated that SESN2 knockdown switched SESN2, p‐mTOR, HIF‐1α, HK2, PFKM, PKM1, PKM2, and GLUT1 protein levels in the BV2 cells after OGD/R. (B–I) Quantitative analysis of SESN2, p‐mTOR, HIF1‐α, HK2, PFKM, PKM1, PKM2, and GLUT1 levels in the BV2 cells after OGD/R. (J) Lactate kit test showed that SESN2 knockdown increased lactate expression in the BV2 cells after OGD/R. (K–M) ELISA indicated that SESN2 knockdown elevated the levels of inflammatory factors after OGD/R. (N) Schematic diagram of the coculture system of HT22 cells and BV2 cells. (O) CCK‐8 assay analyzed the relative viability of HT22 cells. (P) WB strips and (Q, R) quantitation showed the Cl‐caspase 1 and Cl‐caspase 8 protein levels. *n* = 5. ^ns^ *p* > 0.05, *p < 0.05, **p < 0.01, ***p < 0.001. Means ± SD.

To investigate the effect of SESN2 on microglia metabolism after stroke, lentivirus (Lenti‐SESN2) was subjected to knock down SESN2 in BV2 cells (Figure 1O). WB analysis illustrated that the SESN2 level was decreased in the Lenti‐SESN2 groups (Lenti‐SESN2‐1, Lenti‐SESN2‐2 and Lenti‐SESN2‐3) compared to the Lenti‐GFP group, and the Lenti‐SESN2‐1 had the highest interference efficiency (Figure 1P,Q). We used the Lenti‐SESN2‐1 for subsequent experiments. First, WB illustrated that 24 h after OGD/R, SESN2 knockdown elevated the levels of HK2, PFKM, PKM1, PKM2, and GLUT1 (Figure 2A,B,E–I). Meanwhile, RT‐qPCR revealed a similar result (Figure S1A–F). Additionally, the lactate level was increased after 24 h of OGD/R, and SESN2 knockdown showed a further increased lactate level (Figure 2J).

The mTOR/HIF‐1α pathway is involved in multiple metabolisms including glucose metabolism. We examined the mTOR/HIF‐1α levels in BV2 cells by WB. The results illustrated that the p‐mTOR/HIF‐1α pathway levels were elevated 24 h after OGD/R and were further elevated with SESN2 knockdown, indicating that the mTOR/HIF‐1α pathway was further activated after SESN2 knockdown (Figure 2A–D).

### 3.3 SESN2 Knockdown Enhanced Neuroinflammation and Promoted Neuronal Damage In Vitro

Changes in glucose metabolism can cause alterations of immune cell function [30]. To investigate the role of SESN2 in microglial function, RT‐qPCR and Elisa assay were used to examine the pro‐inflammatory factors (IL‐6, IL‐1β, and TNF‐α) levels in BV2 cells. The results showed that the pro‐inflammatory factors levels were elevated after OGD/R. However, SESN2 knockdown further elevated the pro‐inflammatory factors levels (Figure 2K–M). These revealed that SESN2 knockdown enhances the neuroinflammation (Figure S1G–J).

Neuroinflammation is an important cause of secondary neural damage after IS [25, 30]. We further investigated the role of SESN2 in neuronal damage, and a microglia–neuron (BV2‐HT22) coculture system was applied (Figure 2N). CCK‐8 assays illustrated that 24 h after OGD/R injury, SESN2 knockdown in BV2 cells dramatically decreased the viability of HT22 cells (Figure 2O). Moreover, WB analysis showed that SESN2 knockdown in BV2 cells elevated the CL‐caspase 1 and CL‐caspase 8 levels in HT22 cells (Figure 2P–R). These suggested that SESN2 knockdown promoted neuronal damage in vitro.

### 3.4 Knockdown of SESN2 Exacerbated I/R Injury and Neuroinflammation in Mice

**FIGURE 3:**
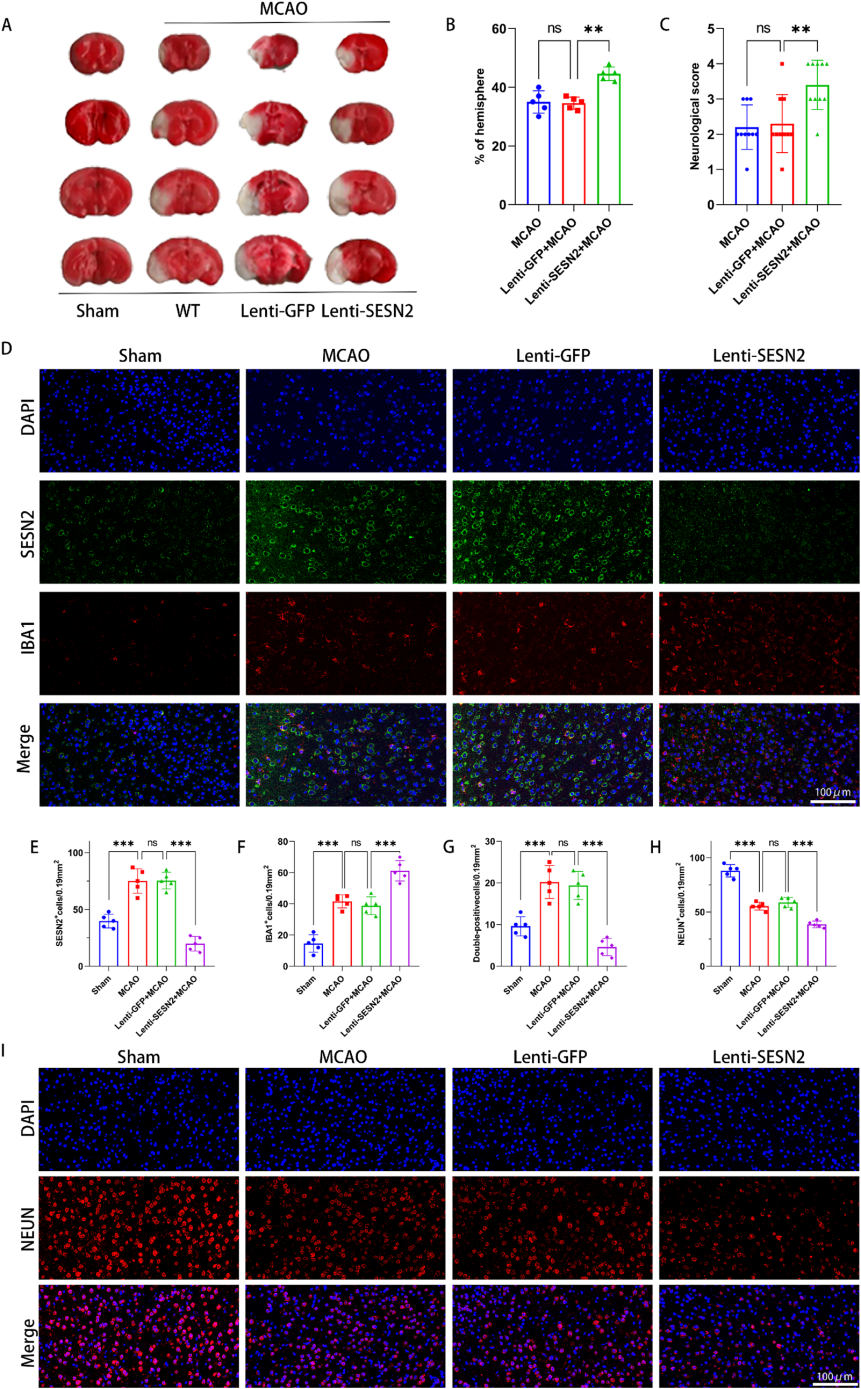
Sesn2 knockdown aggravated brain injury and neurological deficits 72 h after I/R. (A) TTC‐stained sections showing infarcts in mice brain. (B) Quantitative analyses of the infarct volume after 72 h of stroke. (C) Statistical analysis of neurological scores 3 days after I/R. *n* = 10. (D) Representative images of immunostaining showed that SESN2 knockdown elevated the IBA1^+^ cell counts in the peri‐ischemic region after 72 h of stroke. (E–G) Statistical analysis of the SESN2^+^, IBA1^+^, and double‐positive cell counts. (H) Statistical analysis of NEUN^+^ cells. (I) Representative images of immunostaining illustrated that SESN2 knockdown declined the NEUN^+^ cell counts 72 h after stroke. *n* = 5. ^ns^ *p* > 0.05, **p < 0.01, ***p < 0.001. Means ± SD. Scale bar = 100 μm.

**FIGURE 4:**
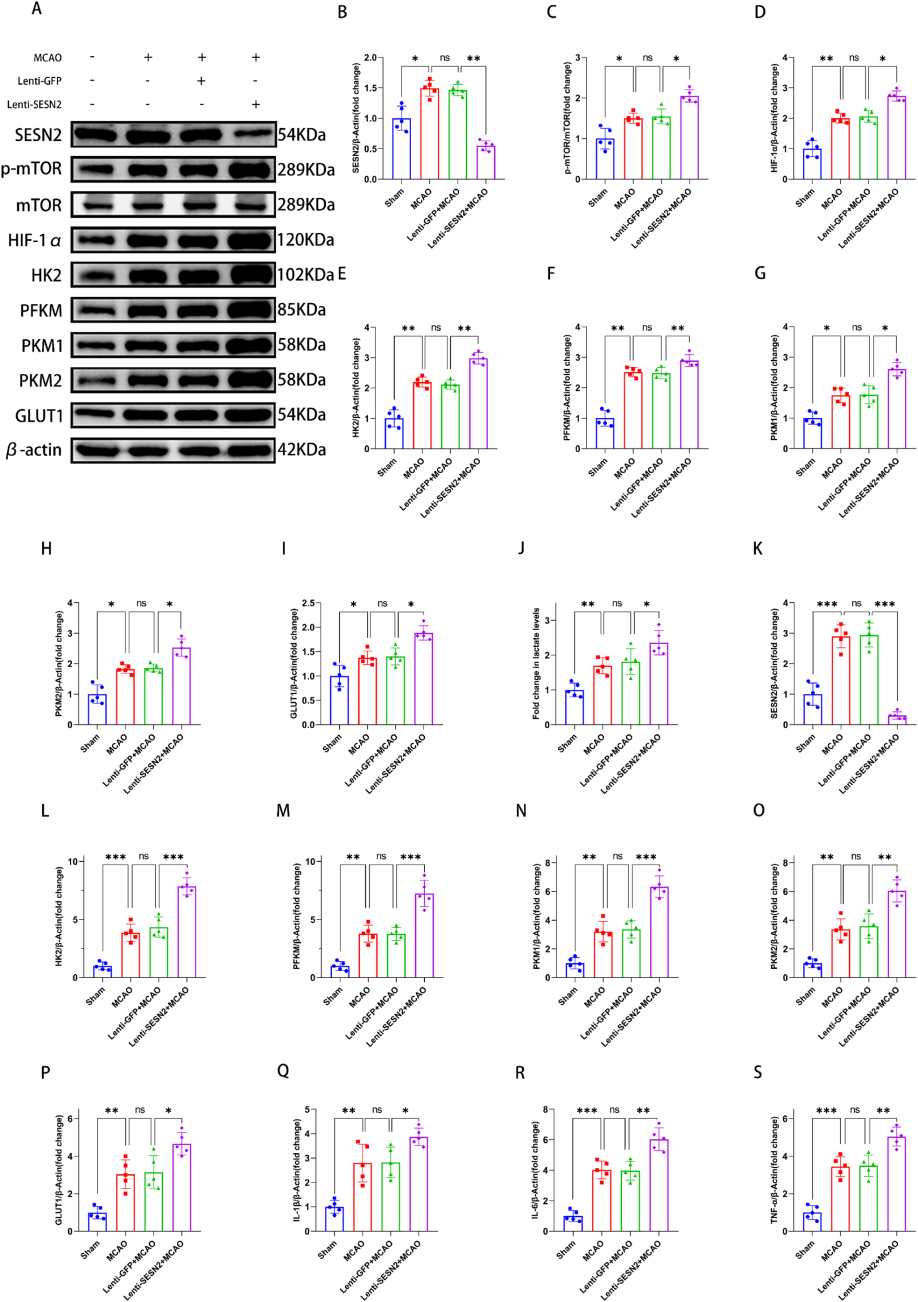
Sesn2 knockdown promoted glycolysis and p‐mTOR/HIF‐1α pathway in ischemic brain after 3 days of stroke. (A) Western blot strips showed that SESN2 knockdown switched the expression of SESN2, p‐mTOR, HIF‐1α, HK2, PFKM, PKM1, PKM2, and GLUT1 in the peri‐ischemic region 72 h after I/R. (B–I) Quantitation of SESN2, HIF‐1α, p‐mTOR, HK2, PFKM, PKM1, PKM2, and GLUT1 levels. (J) Lactate kit test indicated that SESN2 knockdown increased lactate levels after 3 days of I/R. (K–P) RT‐qPCR indicated that SESN2 knockdown increased the mRNA levels of HK2, PFKM, PKM1, PKM2, and GLUT1. (Q–S) RT‐qPCR indicated that SESN2 knockdown increased the mRNA levels of pro‐inflammatory factors. *n* = 5. ^ns^ *p* > 0.05, **p < 0.05, **p < 0.01, ***p < 0.001. Means ± SD.

Then, we constructed an IS model after treating mice with Lenti‐SESN2 or Lenti‐GFP for 5 days. IF staining showed that Lenti‐SESN2 treatment knocked down the expression of SESN2 after I/R injury (Figure 3D,E). Then, at 3 days after I/R, compared with the Lenti‐GFP treatment group, SESN2 knockdown significantly increased the infarct volume and neurological deficit, and the NEUN^+^ cell counts were decreased in the Lenti‐SESN2 group (Figure 3A–C,I,H). Besides, the knockdown of SESN2 also increased the IBA1^+^ cells in the peri‐ischemic region (Figure 3D,F,G). Furthermore, Elisa assay showed that pro‐inflammatory factor levels were elevated in the brain after 3 days of I/R injury. The knockdown of SESN2 further increased the pro‐inflammatory factor levels (Figure 4Q–S, Figure S1K). These suggested that knockdown of SESN2 exacerbated I/R Injury and neuroinflammation in mice.

### 3.5 Knockdown of SESN2 Enhanced Glycolysis and Activated the mTOR/HIF‐1α Pathway 3 Days After MCAO

**FIGURE 5:**
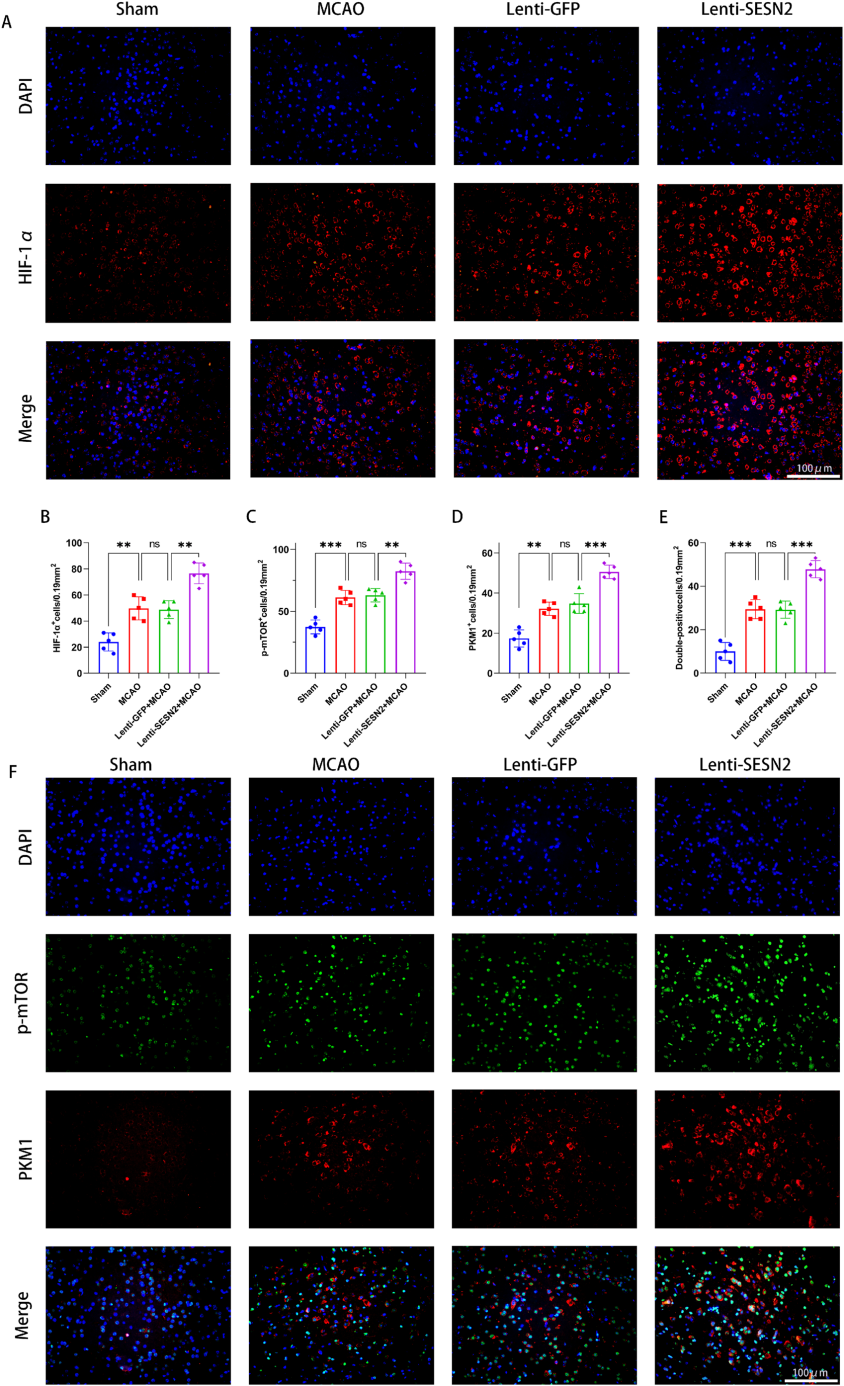
Sesn2 knockdown promoted glycolysis and p‐mTOR/HIF‐1α pathway in ischemic brain 72 h after stroke. (A) Representative images of immunostaining showed that SESN2 knockdown elevated the number of HIF‐1α^+^ cells after 3 days of I/R. (B) Statistical analysis of HIF‐1α^+^ cell counts. (C, D) Statistical analysis of the p‐mTOR^+^ and PKM1^+^ cell counts. (E) Statistical analysis of double‐positive cell counts. (F) Immunostaining showed that SESN2 knockdown increased the p‐mTOR^+^ cell and PKM1^+^ cell counts. *n* = 5. ^ns^ *p* > 0.05, **p < 0.01, ***p < 0.001. Means ± SD. Scale bar = 100 μm.

We examined the levels of glycolysis‐related protein and lactate in mice. WB revealed that SESN2 knockdown upregulated the expression of HK2, PFKM, PKM1, PKM2, and GLUT1 and increased the level of lactate after MCAO (Figure 4A,B,E–J). Meanwhile, RT‐qPCR revealed a similar result (Figure 4K–P). Besides, WB revealed that SESN2 knockdown elevated the p‐mTOR/mTOR and HIF‐1α (Figure 4A–D). Moreover, IF staining illustrated that the SESN2 knockdown elevated the number of PKM1‐positive, HIF‐1α‐positive, and p‐mTOR‐positive cells (Figure 5). These indicated that the knockdown of SESN2 enhanced glycolysis and the mTOR/HIF‐1α pathway in I/R‐induced mice.

### 3.6 SESN2 Regulates Microglial Glycolysis via the mTOR/HIF‐1α Pathway After I/R Injury

**FIGURE 6:**
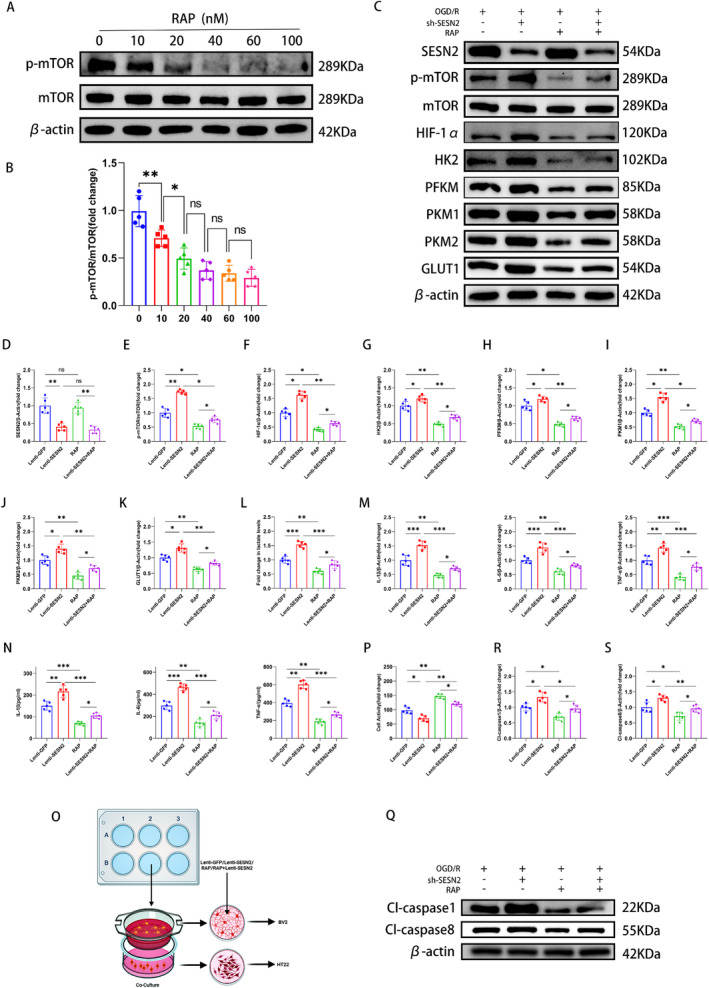
RAP suppressed glycolysis via p‐mTOR/HIF‐1α pathway in the BV2 cells after 24 h of OGD/R. (A) Western blot strips illustrated that RAP inhibited the p‐mTOR protein levels in the BV2 cells. (B) Quantification of p‐mTOR level in the BV2 cells. (C) Western blot strips illustrated that RAP suppressed the p‐mTOR, HIF‐1α, HK2, PFKM, PKM1, PKM2, and GLUT1 protein levels in the BV2 cells after 24 h of OGD/R. (D–K) Quantitative analysis of SESN2, p‐mTOR, HIF1‐α, HK2, PFKM, PKM1, PKM2, and GLUT1 levels in BV2 cells. (L) Lactate kit test illustrated that rapamycin suppressed the lactate production in the BV2 cells after OGD/R. (M) RT‐qPCR and (N) ELISA showed that rapamycin treatment decreased the levels of pro‐inflammatory factors. (O) Schematic diagram of the coculture system of HT22 cells and BV2 cells. (P) CCK‐8 assay analyzed the relative viability of HT22 cells. (Q) Western blot strips illustrated the apoptosis‐related proteins levels. (R, S) Quantitation of Cl‐caspase 1 and Cl‐caspase 8 levels. *n* = 5. ^ns^ *p* > 0.05, *p < 0.05, **p < 0.01, ***p < 0.001. Means ± SD.

To investigate whether SESN2 mediates microglial glycolysis after I/R injury through the mTOR/HIF‐1α pathway, we treated OGD/R‐induced BV2 cells and IS mice with rapamycin. According to the result of WB analysis, we selected 20 nM rapamycin for subsequent cell experiments (Figure 6A,B). In vitro, the results illustrated that after 24 h of OGD/R, rapamycin treatment decreased the levels of p‐mTOR, HIF‐1α, HK2, PFKM, PKM1, PKM2, and GLUT1 in BV2 cells (Figure 6C–K). However, rapamycin treatment did not affect the level of SESN2 (Figure 6C–K). Besides, rapamycin treatment also decreased the lactate and inflammatory factors levels in OGD/R‐induced BV2 cells with SESN2 knockdown (Figure 6L–N). Furthermore, CCK8 assay showed that rapamycin treatment could partially reverse the HT22 cell injury caused by SESN2 knockdown in BV2 cells under the OGD/R condition (Figure 6O,P). Meanwhile, WB revealed a similar result (Figure 6Q–S).

In vivo, we validated the critical role of the mTOR/HIF‐1α pathway using rapamycin (10 mg/kg, i.p.). In SESN2 knockdown mice, rapamycin treatment reduced the neurological score and infarct volume; decreased the p‐mTOR, HIF‐1α, HK2, PFKM, PKM1, PKM2, and GLUT1; and lowered the lactate level 3 days after MCAO (Figure S2A–M). Meanwhile, rapamycin treatment decreased the expression of inflammatory factors in SESN2 knockdown IS mice (Figure S2N–P).

These results illustrated that SESN2 knockdown exacerbated I/R injury by promoting glycolysis via the mTOR/HIF‐1α pathway.

## 4 Discussion

In this research, we found that the knockdown of SESN2 exerted a nerve‐damaging effect by promoting the mTOR/HIF‐1α pathway. The application of SESN2 lentivirus could significantly increase the infarct size and neurological deficit 3 days after MCAO. Moreover, the knockdown of SESN2 promoted the expression of glycolysis‐related proteins, and various mRNA levels, and lactate levels. Moreover, we demonstrated that cerebral ischemia–reperfusion injury (CIRI) promoted mTOR/HIF‐1α signaling pathway expression, which could be further enhanced via the knockdown of SESN2. However, the inhibition of mTOR reduced the expression of glycolysis‐related proteins and lactate levels. These results indicate that mTOR promotion by the knockdown of SESN2 in MCAO mice and OGD BV2 cells enhanced HIF‐1α expression levels, which in turn enhance the downstream glycolysis‐related proteins and lactate levels, leading to increased glycolysis activation and aggravated damage.

As a stress‐induced protein, SESN2 plays an essential role in cellular adaptation to stress states due to its anti‐reactive oxygen species (anti‐ROS), metabolic regulation, anti‐inflammatory functions [9, 31, 32, 33], and involvement in various hypoxia‐related diseases [34, 35], and is also expressed in a variety of ischemic tissues such as the ischemic cerebrum. Consistent with these results, SESN2 increased significantly during cerebral ischemia. In addition, SESN2 is involved in the modulation of many important cellular processes, including autophagy [36], apoptosis [37], metabolic reprogramming [38], and pyroptosis [39]. Moreover, recent research has confirmed that SESN2 is strongly associated with glucose metabolism in a variety of diseases [40]. The key that SESN2 can regulate glucose metabolism reprogramming lies in its ability to regulate metabolism‐related pathways, such as mTOR and AMPK [41, 42]. Glucose metabolism reprogramming (transition from oxidative phosphorylation to glycolysis) after I/R can aggravate CIRI; however, CIRI can be improved by inhibiting this metabolic reprogramming [7, 8]. As a glycolytic intermediate, lactate plays an important role in energy metabolism by providing a convenient three‐carbon source and sink and balancing the ratio of NADH/NAD between cells and tissues [43, 44]. However, in ischemic diseases such as cerebral ischemia and hypoxia (HI) [45], an important reason for the aggravation of damage caused by enhanced glycolysis is the increased flux of glycolysis to lactate production. Moreover, it has been shown that glycolysis and lactate levels are closely correlated with the levels of inflammation, while high levels of glycolysis and lactate are often accompanied by increased levels of inflammation [46, 47]. However, the metabolic changes in glucose metabolism after IS have not been studied clearly, and the mechanisms remain to be elucidated. Here, we show for the first time that knockdown of SESN2 increased glucose transporter‐1 (GLUT1) levels; increased many key glycolytic enzyme levels, such as pyruvate kinase M1 (PKM1), hexokinase 2 (HK2), phosphofructokinase‐1 (PFKM), pyruvate kinase M2 (PKM2); and in turn increased lactate levels. Moreover, the levels of pro‐inflammatory factors were further increased after SESN2 knockdown. Furthermore, knockout of SESN2 in microglia in vitro increased the level of apoptosis in cocultured neurons. These results indicated that knockdown of SESN2 increased glycolysis and lactate levels, which in turn aggravated post‐stroke inflammation and increased cell apoptosis, leading to nerve damage.

The mechanism by which SESN2 regulates glucose metabolism reprogramming is unclear, and a few studies have been reported. In the previous discussion, SESN2 may protect brain tissue to mitigate reperfusion injury by suppressing the expression level of mTOR/HIF‐1α and then downregulating the glycolysis‐related proteins and lactate expression levels. The mTOR/HIF‐1α pathway is strongly associated with glucose metabolism in a variety of diseases such as Alzheimer's and tumors [48]. As a downstream factor of mTOR, HIF‐1α is an essential regulator of glycolysis, which regulates glucose metabolism by activating some of the key enzymes in glucose metabolism [49, 50]. Accumulating evidence suggests that the mTOR/HIF‐1α pathway is associated with ischemic diseases such as ischemic myocardial infarction due to its pro‐angiogenic effect [51]. However, it is unclear whether mTOR/HIF‐1α is involved in glucose metabolism reprogramming after IS and whether SESN2 affects CIRI through regulating glucose metabolism reprogramming via mTOR/HIF‐1α. This study demonstrated that after 3 days of I/R, the SESN2 and mTOR/HIF‐1α signaling pathway levels were enhanced. The glycolysis‐related proteins and lactate levels were also significantly increased. In contrast, the mTOR/HIF‐1α levels were significantly upregulated with SESN2 silencing, and the aerobic glycolytic protein and lactate expression were also further increased with SESN2 silencing. However, all of these effects of the SESN2 knockdown were reversed after the application of the mTOR inhibitor rapamycin. These results once again confirm our speculation. This work has some limitations. We focused on the glucose metabolism reprogramming effects of SESN2 rather than its other effects, such as its angiogenesis and anti‐inflammatory effects [52]. We focused on glucose metabolism reprogramming in BV2 cells in vitro, but neurons may also undergo glucose metabolism reprogramming in vitro. Moreover, how increased lactate production caused by the knockdown of SESN2 affects stroke outcome needs further elucidation, which can be investigated by examining the corresponding sites of histone lactationization. Therefore, the other potential mechanisms underlying the neuroprotective effects of SESN2 in CIRI, including the role in neuroinflammation and histone lactationization, need to be further elucidated.

## 5 Conclusion

In summary, our results suggest that the knockdown of SESN2 promotes glycolysis through the activation of the mTOR/HIF‐1α signaling pathway, which increases lactate production and thus exhibits neurological damaging effects on CIRI.

## Conflicts of Interest

The authors declare no conflicts of interest.

## Supporting information


Data S1.

**Figure S1.** Effect of SESN2 knockdown on glycolysis‐related proteins expression and inflammation. (A–F) RT‐qPCR indicated that SESN2 knockdown increased the mRNA levels of HK2, PFKM, PKM1, PKM2, and GLUT1 after OGD/R. (G–J) RT‐qPCR indicated that SESN2 knockdown elevated the levels of inflammatory factors after OGD/R. (K) RT‐qPCR indicated that SESN2 knockdown decreased the levels of anti‐inflammatory factors after MCAO. *n* = 5. ^ns^ *p* > 0.05, *p < 0.05, **p < 0.01, ***p < 0.001. Means ± SD.
**Figure S2.** RAP suppressed glycolysis via p‐mTOR/HIF‐1α pathway in ischemic brain 72 h after stroke. (A) TTC‐stained sections showing infarcts in each group of mice. (B) Quantitative analyses of the infarct volume after 72 h of MCAO. (C) Statistical analysis of neurologic scores 3 days after I/R. *n* = 10. (D) Western blots strips illustrated that RAP suppressed the p‐mTOR, HIF‐1α, HK2, PFKM, PKM1, PKM2, and GLUT1 protein levels in the peri‐ischemic region after stroke. (E–L) Quantitation of SESN2, p‐mTOR/HIF‐1α, and glycolysis‐related proteins. (M) Lactate kit test showed that rapamycin treatment decreased the levels of lactate 72 h after I/R. (N–P) RT‐qPCR showed that rapamycin treatment suppressed the pro‐inflammatory factors levels 72 h after I/R. *n* = 5. ^ns^ *p* > 0.05, *p < 0.05, **p < 0.01, ***p < 0.001. Means ± SD.

## Data Availability

The data that support the findings of this study are available from the corresponding author upon reasonable request.
