# Supplementary figures and images for: Knockdown of SESN2 Exacerbates Cerebral Ischemia–Reperfusion Injury Through Enhancing Glycolysis via the mTOR/HIF‐1α Pathway
Source: CNS Neurosci Ther. 2025 Mar 3;31(3):e70314. doi: 10.1111/cns.70314 (PMC11875773; doi:10.1111/cns.70314)

Figure1

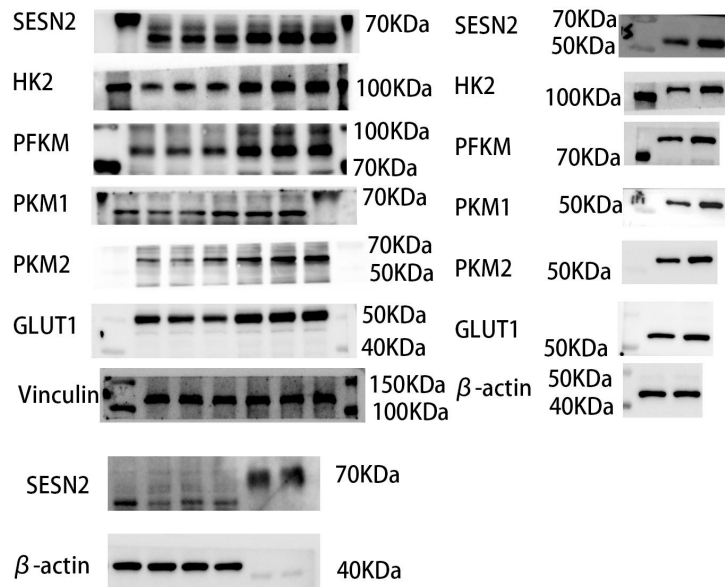

Figure4

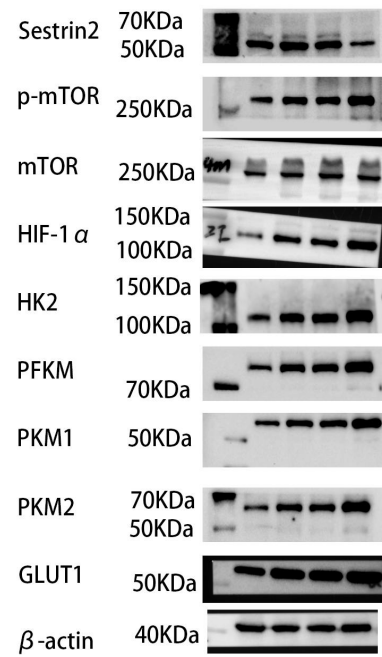

Figure2

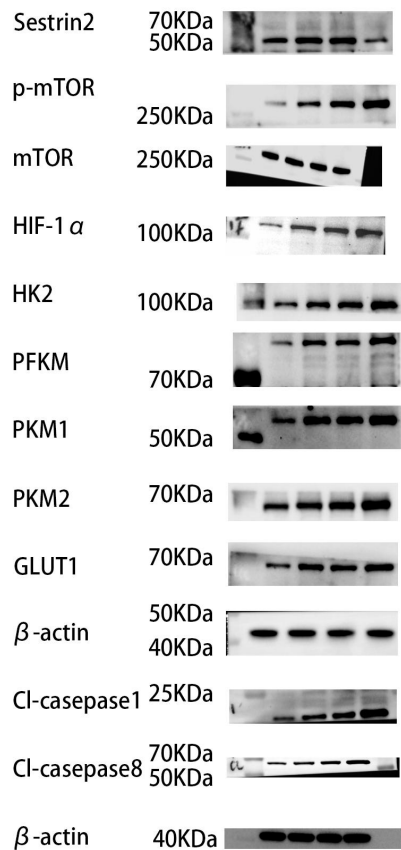

Figure6

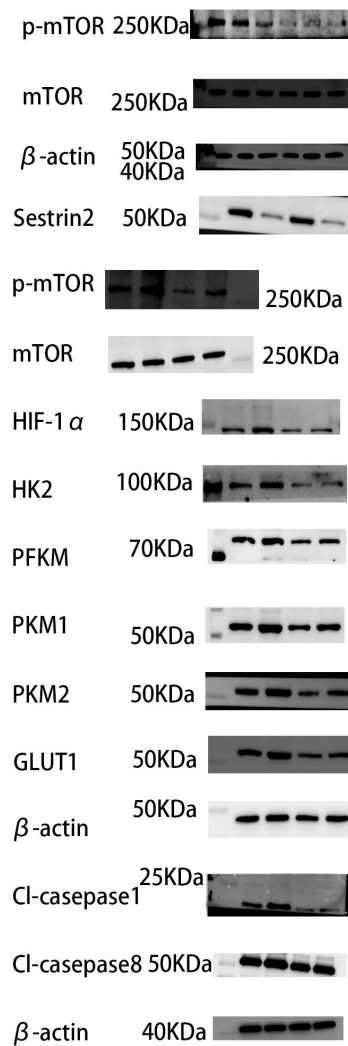

Supplemental Figure 2

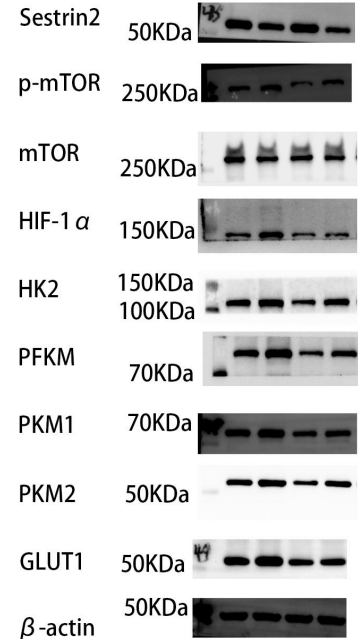

Supplement: Supplementary file 1 — Data S1. [file CNS-31-e70314-s001.pdf]
