# Supplementary material for: Knockdown of SESN2 Exacerbates Cerebral Ischemia–Reperfusion Injury Through Enhancing Glycolysis via the mTOR/HIF‐1α Pathway
Source: CNS Neurosci Ther. 2025 Mar 3;31(3):e70314. doi: 10.1111/cns.70314 (PMC11875773; doi:10.1111/cns.70314)
Supplement: Supplementary file 2 — Figure S1. Effect of SESN2 knockdown on glycolysis‐related proteins expression and inflammation. (A–F) RT‐qPCR indicated that SESN2 knockdown increased the mRNA levels of HK2, PFKM, PKM1, PKM2, and GLUT1 after OGD/R. (G–J) RT‐qPCR indicated that SESN2 knockdown elevated the levels of inflammatory factors after OGD/R. (K) RT‐qPCR indicated that SESN2 knockdown decreased the levels of anti‐inflammatory factors after MCAO. n = 5. ns p > 0.05, *p < 0.05, **p < 0.01, ***p < 0.001. Means ± SD. Figure S2. RAP suppressed glycolysis via p‐mTOR/HIF‐1α pathway in ischemic brain 72 h after stroke. (A) TTC‐stained sections showing infarcts in each group of mice. (B) Quantitative analyses of the infarct volume after 72 h of MCAO. (C) Statistical analysis of neurologic scores 3 days after I/R. n = 10. (D) Western blots strips illustrated that RAP suppressed the p‐mTOR, HIF‐1α, HK2, PFKM, PKM1, PKM2, and GLUT1 protein levels in the peri‐ischemic region after stroke. (E–L) Quantitation of SESN2, p‐mTOR/HIF‐1α, and glycolysis‐related proteins. (M) Lactate kit test showed that rapamycin treatment decreased the levels of lactate 72 h after I/R. (N–P) RT‐qPCR showed that rapamycin treatment suppressed the pro‐inflammatory factors levels 72 h after I/R. n = 5. ns p > 0.05, *p < 0.05, **p < 0.01, ***p < 0.001. Means ± SD. [file CNS-31-e70314-s002.docx]

Supplemental Figures


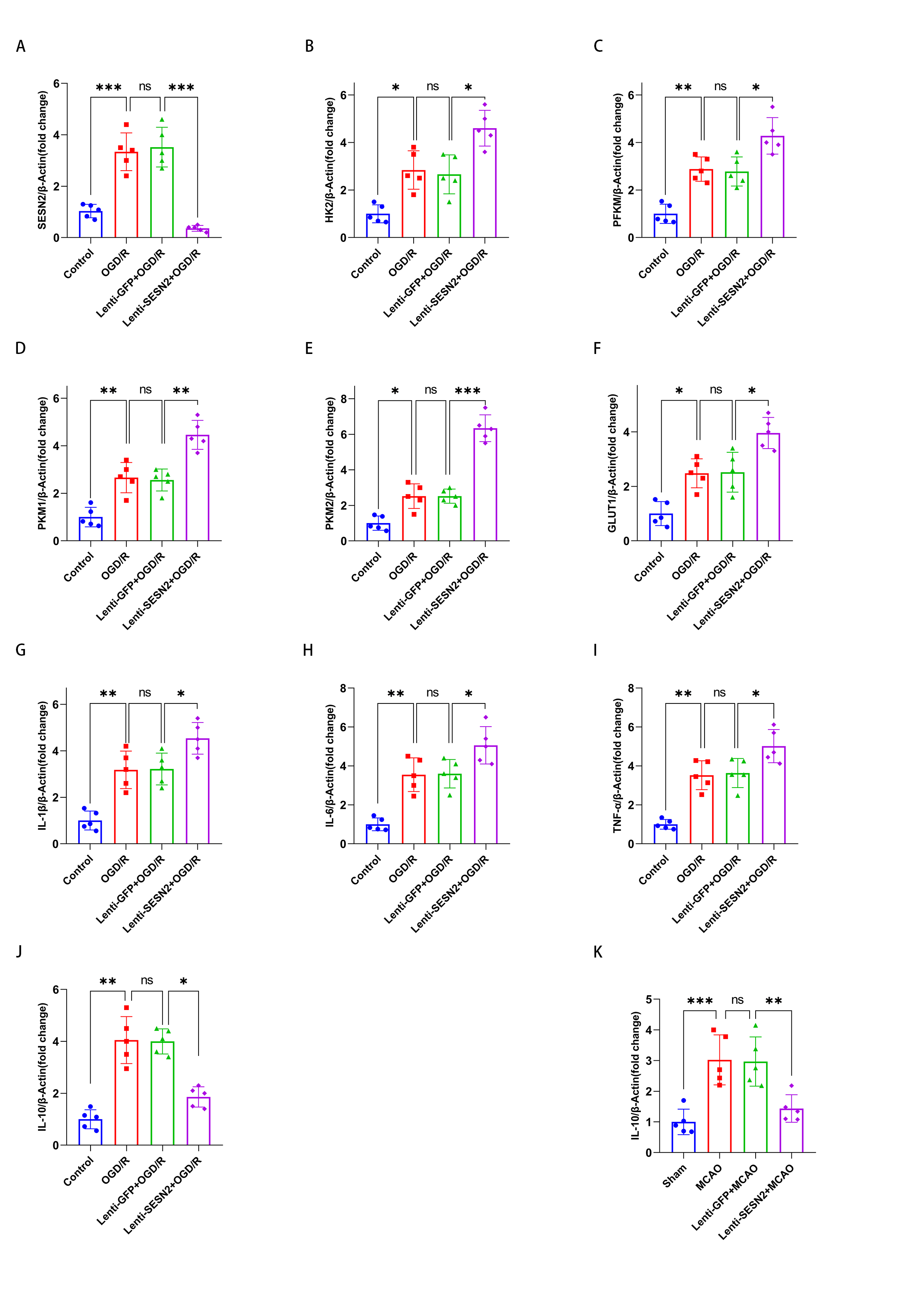
Supplemental Figure 1. Effect of SESN2 knockdown on glycolysis-related proteins expression and inflammation. (A-F) RT-qPCR indicated that SESN2 knockdown increased the mRNA levels of HK2, PFKM, PKM1, PKM2 and GLUT1 after OGD/R. (G-J) RT-qPCR indicated that SESN2 knockdown elevated the levels of inflammatory factors after OGD/R. (K) RT-qPCR indicated that SESN2 knockdown decreased the levels of anti-inflammatory factors after MCAO. n = 5. ^ns^ p>0.05, * p < 0.05, ** p < 0.01, *** p < 0.001. Means ± SD.


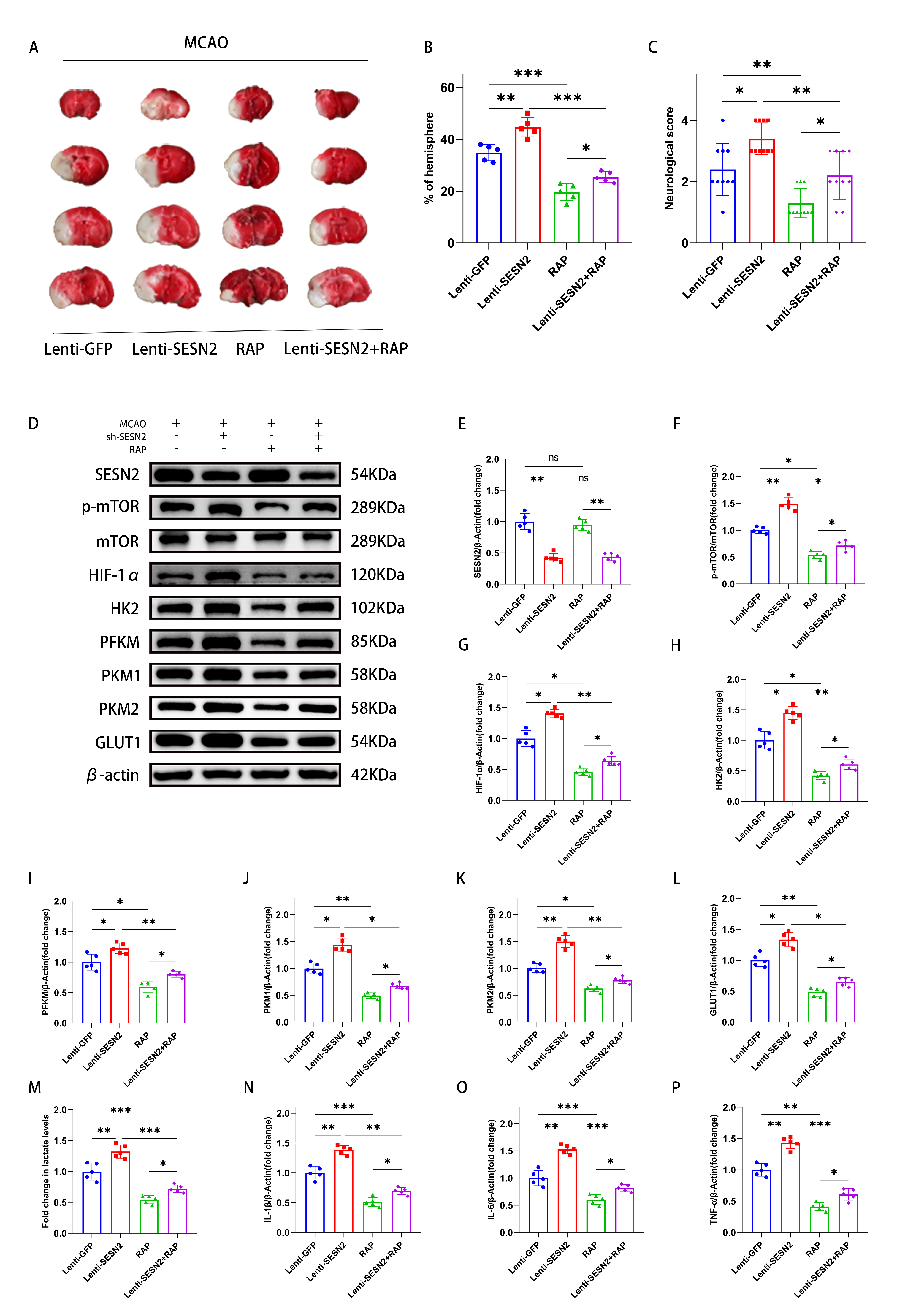


Supplemental Figure 2. RAP suppressed glycolysis via p-mTOR/HIF-1α pathway in ischemic brain 72 h after stroke. (A) TTC-stained sections showing infarcts in each group of mice. (B) Quantitative analyses of the infarct volume after 72 h of MCAO. (C) Statistical analysis of neurologic scores 3 days after I/R. n = 10. (D) Western blots strips illustrated that RAP suppressed the p-mTOR, HIF-1α, HK2, PFKM, PKM1, PKM2 and GLUT1 protein levels in the peri-ischemic region after stroke. (E-L) Quantitation of SESN2, p-mTOR/HIF-1α and glycolysis-related proteins. (M) Lactate kit test showed that rapamycin treatment decreased the levels of lactate 72 h after I/R. (N-P) RT-qPCR showed that rapamycin treatment suppressed the pro-inflammatory factors levels 72 h after I/R. n = 5. ^ns^ p>0.05, * p < 0.05, ** p < 0.01, *** p < 0.001. Means ± SD.
